# Increased Beta-Hydroxybutyrate Level Is Not Sufficient for the Neuroprotective Effect of Long-Term Ketogenic Diet in an Animal Model of Early Parkinson’s Disease. Exploration of Brain and Liver Energy Metabolism Markers

**DOI:** 10.3390/ijms22147556

**Published:** 2021-07-14

**Authors:** Katarzyna Z. Kuter, Łukasz Olech, Urszula Głowacka, Martyna Paleczna

**Affiliations:** Deptartment of Neuropsychopharmacology, Maj Institute of Pharmacology Polish Academy of Sciences, Smętna 12 St., 31-343 Krakow, Poland; lukasz_olech@interia.pl (Ł.O.); urszula.d86@vp.pl (U.G.); paleczna@if-pan.krakow.pl (M.P.)

**Keywords:** liver gluconeogenesis, glycogen, mitochondrial complex II, neurodegeneration, compensation, substantia nigra, beta-hydroxybutyrate, ketone bodies

## Abstract

The benefits of a ketogenic diet in childhood epilepsy steered up hope for neuroprotective effects of hyperketonemia in Parkinson’s disease (PD). There are multiple theoretical reasons but very little actual experimental proof or clinical trials. We examined the long-term effects of the ketogenic diet in an animal model of early PD. A progressive, selective dopaminergic medium size lesion was induced by 6-OHDA injection into the medial forebrain bundle. Animals were kept on the stringent ketogenic diet (1% carbohydrates, 8% protein, 70% fat) for 3 weeks prior and 4 weeks after the brain operation. Locomotor activity, neuron count, dopaminergic terminal density, dopamine level, and turnover were analyzed at three time-points post-lesion, up to 4 weeks after the operation. Energy metabolism parameters (glycogen, mitochondrial complex I and IV, lactate, beta-hydroxybutyrate, glucose) were analyzed in the brain and liver or plasma. Protein expression of enzymes essential for gluconeogenesis (PEPCK, G6PC) and glucose utilization (GCK) was analyzed in the liver. Despite long-term hyperketonemia pre- and post-lesion, the ketogenic diet did not protect against 6-OHDA-induced dopaminergic neuron lesions. The ketogenic diet only tended to improve locomotor activity and normalize DA turnover in the striatum. Rats fed 7 weeks in total with a restrictive ketogenic diet maintained normoglycemia, and neither gluconeogenesis nor glycogenolysis in the liver was responsible for this effect. Therefore, potentially, the ketogenic diet could be therapeutically helpful to support the late compensatory mechanisms active via glial cells but does not necessarily act against the oxidative stress-induced parkinsonian neurodegeneration itself. A word of caution is required as the stringent ketogenic diet itself also carries the risk of unwanted side effects, so it is important to study the long-term effects of such treatments. More detailed metabolic long-term studies using unified diet parameters are required, and human vs. animal differences should be taken under consideration.

## 1. Introduction

Parkinson’s disease (PD) is a slowly progressing neurodegenerative disorder that shows its first significant motor symptoms at alate age [1]. Interestingly, at the early PD stages, until the degeneration is very advanced, only diffuse, often peripheral problems in patients’ health are observed. The pre-clinical stage of PD is established to last many years before an approximate 70% decrease of dopaminergic neurons in the substantia nigra (SN) and 80% loss of dopamine (DA) in its output structure—striatum (STR)—resulting in clear motor dysfunction. Thus far, the cause of neurodegeneration in PD is still unclear, and no neuroprotective treatment is available for patients. However, such a long period of relatively normal functioning gives an opportunity for the introduction of potential neuroprotective treatments.

Among many possible pathologic mechanisms, energy metabolism was described to be disturbed in PD. Dysregulation of energy-sensing pathways was recognized already as an early disease event [2]. Mitochondrial quality control and protein import, redox homeostasis, and central carbon metabolism were described to be affected in PD progression [3,4,5].

The concept of ketogenic, low-carbohydrate/high-fat diet use has been promoted in the past years as a way of mimicking beneficiary fasting effects. Although the exact mechanisms are still unknown, the ketogenic diet has proven functional efficiency in the management of drug-resistant epilepsy, especially in children. Therefore, it has been suggested that other neurological diseases such as PD might also respond well to such treatment. A single published, small clinical study (five PD patients) [6] steered up the hope for such neuroprotective treatment in PD [7]. Although there are multiple reviews on possible therapeutic mechanisms of ketone bodies that could be beneficial in PD [8,9,10,11,12,13,14,15,16,17,18,19,20] there are only four relevant in vivo experimental researches published [21,22,23,24] (Table S1). Those studies indeed showed improved functioning and/or neuroprotective effects of variable forms of ketogenic diets [22,23,24] or ketone body supplementation [21] in animal models of PD. On the other hand, a more recent clinical trial and the only available so far, on 38 PD patients, showed that both ketogenic as well as a low-fat/high-carbohydrate diet improved motor activity and non-motor symptoms, while the ketogenic diet showed even intermittent exacerbation of the tremor and/or rigidity [25], indicating that it is not necessarily the ketone bodies per se that are required for a beneficial diet effect. Further studies showed that the ketogenic diet did not affect the pharmacokinetics of the anti-parkinsonian drug L-DOPA [26]. Dietary interventions seem a very appealing, relatively easy, and applicable way of potential neuroprotective treatment. It is very popular in society, generating multiple non-scientific beliefs. Therefore, a reliable explanation of ketogenic diet effectiveness in PD treatment is worth the professional examination.

The process of ketogenesis involves adipocytes, which break down triglycerides into glycerol, fatty acids, and hepatocytes, which transform the fatty acids into ketone bodies via beta-oxidation. During the ketogenic diet or prolonged fasting, massive secretion of ketone bodies is a pivotal metabolic process to ensure adequate fuel supply for the brain and other tissues [11]. In normally fed adult mammals, metabolism of the main ketone body, β-hydroxybutyrate (b-HB), comprises <3% of total cerebral metabolism and is present in low circulating concentrations (0.1 mmol/L) [27,28]. Due to the ketogenic diet, plasma ketone levels can be increased four- to fivefold within 2 days. Ketone bodies are the natural alternative substrate to glucose for cerebral energy metabolism [11,29]. When glucose availability is scarce, they provide a more efficient energy source and are metabolized faster due to the bypass of the glycolytic pathway and directly entering the tricarboxylic acid (TCA) cycle [30,31]. Consequently, ketone bodies lead to a decrease in glycolytic ATP generation and an increase in ATP generation by mitochondrial oxidation [32,33]. Therefore, the ketogenic diet induces a shift in energy metabolism substrate use. b-HB has been shown to help antioxidant defense and reduce the production of reactive oxygen species (ROS) by improving mitochondrial respiration and bypassing complex (Cx) I dysfunction. Ketogenic diet and/or ketone bodies were shown to act as anti-inflammatories, decrease microglia activation, and increase the production of trophic factors [9,10]. Additionally, studies based on the effectiveness of the ketogenic diet in epilepsy and partially in Alzheimer’s disease have proposed that the ketogenic diet may cause modification in synaptic morphology and function, involving ionic channels, glutamatergic transmission, or synaptic vesicular cycling machinery [8].

In relevance to the PD pathophysiology, dopaminergic neurons possess a particularly high metabolic rate due to an autonomous pacemaking activity, which could make them vulnerable to the energy shortage [34]. At the early stages of PD, partial degeneration of dopaminergic neurons is functionally compensated by surviving neurons and supporting glial cells [35,36,37]. It is probable that such compensation puts higher energetic demands on the remaining dopaminergic neurons; hence, one of the possible mechanisms contributing to this process could be enhanced or more efficient energy production. Therefore, having a more energy-efficient fuel source and improved antioxidant protection on a ketogenic diet, endangered dopaminergic neurons could possess a better resistance and adaptive ability to metabolic stress and challenges [8]. Increased ketone bodies availability due to the ketogenic diet treatment could have, in theory, a therapeutic application in PD by supporting compensation and neuroprotection.

Thus far, the few studies on various forms of low-carbohydrate/high-fat, ketogenic diets in PD showed very different results. There are substantial differences between treatment strategies. Many studies were performed in vitro or used ketone bodies substitution instead of diet treatment. The few animal studies showing neuroprotection in PD models did not analyze glucose levels and significantly varied in the diet’s compositions. Most studies were usually short-term and did not analyze the long-term diet side effects or liver functions, which is the most important supplier of the ketone bodies. Moreover, human vs. animal metabolism responses were not studied sufficiently so far.

This is why our aim was to evaluate the long-term effect of the ketogenic diet on the selective dopaminergic neuron degeneration induced by 6-OHDA as well as brain and liver metabolism parameters in an animal model of early PD. We studied the effects of 7 weeks in total of stringent ketogenic diet administered to rats both prior to and after lesion.

## 2. Results

### 2.1. Chronic Ketogenic Diet Strongly Increases b-HB Both in Plasma and Brain but Does Not Decrease Glucose Levels

**Figure 1:**
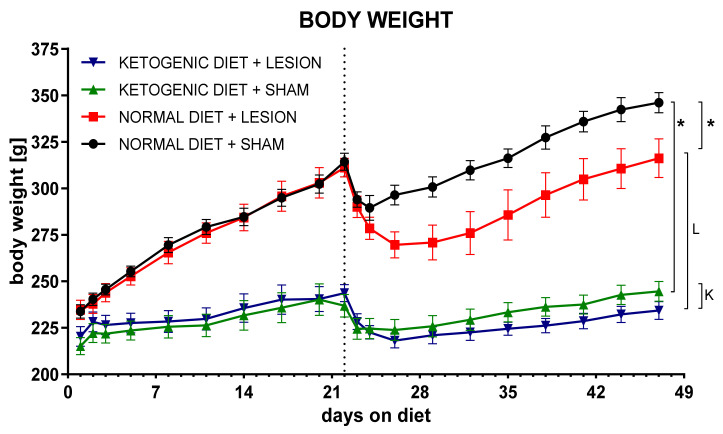
Animal body weight analysis. Data presented as change in body mass, showed as mean ± SEM representative for the group tested for the longest period. Analysis was performed for 3 weeks before and 4 weeks after brain surgery. Dashed line marks the day of operation. *p* value ≤ 0.05 was set as significance threshold and marked as * vs. normal diet + sham, L vs. normal diet + 6-OHDA lesion, K vs. ketogenic diet + sham. Repeated measure ANOVA with LSD post hoc test for analysis in time and 3-way ANOVA with LSD post hoc test for analysis of total values were performed. Number of animals in groups was: for NS *n* = 16; for NL *n* = 14; for KS *n* = 17; for KL *n* = 16.

**Figure 2:**
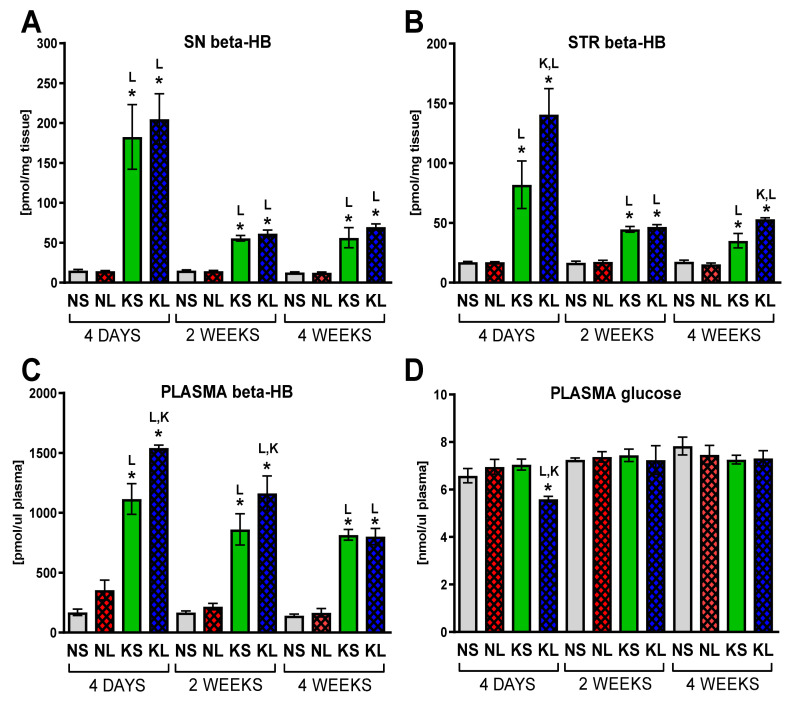
Ketone bodies (**A**–**C**) and glucose (**D**) level analysis. Glucose was measured in plasma and b-HB in plasma and brain homogenates from dopaminergic structures SN and STR. Data are presented as mean ± SEM. Analysis was performed at time-points 4 days, 2 weeks, and 4 weeks after brain surgery. Animals were kept on ketogenic diet for 3 weeks in advance. *p* value ≤ 0.05 was set as significance threshold and marked as * vs. normal diet + sham (NS group), L vs. normal diet + 6-OHDA lesion (NL group), K vs. ketogenic diet + sham (KS group), KL stands for ketogenic diet + 6-OHDA lesion group. Statistics: 3-way ANOVA with LSD post hoc test. Number of animals was 5–7 per group.

Animals were introduced to the strict ketogenic diet (1% carbohydrates, 8% protein, 70% fat, in respect to chow weight) 3 weeks prior to the brain surgery. Still growing, young-adult rats kept on the diet were significantly slower in gaining weight (Figure 1). A ketogenic diet was administered to increase the level of ketone bodies in the blood and brain and switch energy metabolism from glucose to fatty-acid-based. Plasma levels of b-HB increased drastically (very large increases ranging between 566 and 912% of control levels, depending on the treatment and time-point) and brain (an increase of 363–1337% in the SN and in the STR to 199–821% of control, depending on the treatment and time-point) at all analyzed time-points in both groups treated with a ketogenic diet (ketogenic diet + sham (KS) and ketogenic diet + lesion (KL), Figure 2)). Lesioned animals on a ketogenic diet (KL) had significantly higher b-HB levels in plasma 4 days and 2 weeks after surgery and in STR 4 days and 4 weeks, compared to the sham-treated ketogenic diet group (KS). In the SN, b-HB levels did not differ between sham and lesioned groups on the ketogenic diet.

Drastically decreased availability of carbohydrates in the diet up to 7 weeks was supposed to decrease blood glucose levels and, in this way, reprogram the organism to switch to other energy metabolism substrates, such as ketone bodies and fatty acids. Surprisingly, despite this strict diet regimen, the level of plasma glucose was unchanged throughout the whole study period (Figure 2). The only decrease was observed 4 days after surgery in the combined treatment group (KL), probably due to the additive role of diet, decreased locomotion, and smaller food intake after the operation procedure.

### 2.2. Ketogenic Diet Does Not Protect DA Neurons from 6-OHDA Induced Degeneration

**Figure 3:**
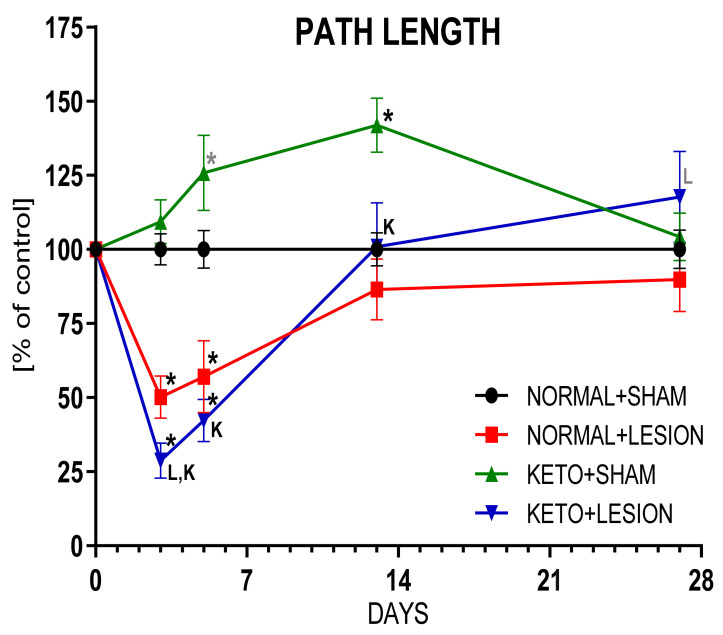
Locomotor activity of rats. Data presented as path length measured in actometers, showed as % of control ± SEM of the respective day. Analysis was performed at time-points 3, 5, 13, 27 days after brain surgery. Animals were kept on ketogenic diet for 3 weeks in advance. *p* value ≤ 0.05 was set as significance threshold and marked as * vs. normal diet + sham, L vs. normal diet + 6-OHDA lesion, K vs. ketogenic diet + sham. Statistical trends 0.05 < *p* ≤ 0.1 were marked in grey symbols. Repeated measure ANOVA with LSD post hoc test for analysis in time and 3-way ANOVA with LSD post hoc test for analysis of total values were performed. Number of animals in groups was: for 3 days, *n* = 19–26; for 5 days, *n* = 8–12; for 13 days, *n* = 9–16; for 27 days, *n* = 8–14.

Ketogenic diet alone significantly increased locomotor activity 5 days and 2 weeks after brain operation (26 and 34 days on diet; *p* = 0.078, *p* = 0.002, respectively), which could be partially a food-seeking behavior, but at 4 weeks post-operation returned to the control levels (Figure 3, Table 1). Injection of 6-OHDA into the MFB fibers, passing from the SN to the STR, causes progressive degeneration of dopaminergic neurons by a dying-back effect [38]. Similar as described in our previous studies [35,39], 3 and 5 days after 6-OHDA injection, rat locomotor activity and the number of rearings were significantly decreased and after 2 weeks returned to the normal values due to the compensatory mechanisms and system plasticity adapting to the medium size of degeneration. Lesioned animals pre-treated by 3 weeks of strict ketogenic diet at first (3 days after the operation) showed pronounced deficits (more decreased path length, rearing time, and number of rearings—free, supported and total, increased resting time), but after 4 weeks, their behavior showed a tendency to improve (*p* = 0.098), compared to 6-OHDA alone group.

**Figure 4:**
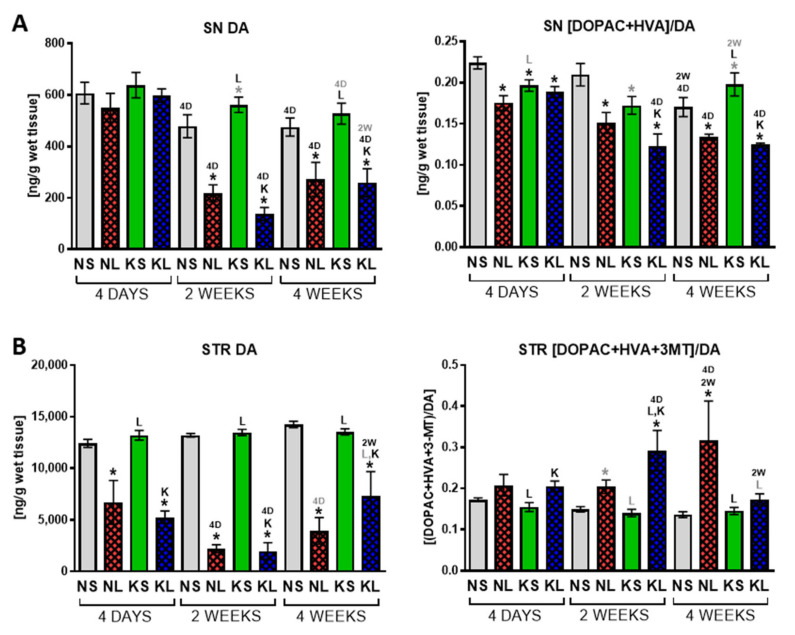
HPLC analysis of DA and its turnover rate in dopaminergic structures SN (**A**) and STR (**B**). Turnover rate was calculated as sum of measured metabolites to neurotransmitter ratio. Data are presented as mean ± SEM. Analysis was performed at time-points 4 days, 2 weeks, and 4 weeks after brain surgery. Animals were kept on ketogenic diet for 3 weeks in advance. *p* value ≤ 0.05 was set as significance threshold and marked as * vs. normal diet + sham (NS group), L vs. normal diet + 6-OHDA lesion (NL group), K vs. ketogenic diet + sham (KS group), KL stands for ketogenic diet + 6-OHDA lesion group, 2W vs. 2 week time-point, 4D vs. 4 day time-point. Statistical trends 0.05 < *p* ≤ 0.1 were marked in grey symbols. Statistics: 3-way ANOVA with LSD post hoc test. Number of animals was 5–7 per group.

HPLC analysis of the neurotransmitter tissue concentration (Figure 4, Table S2) showed that the ketogenic diet had no influence on DA level and its metabolism in the SN or STR after dopaminergic system lesion. The changes observed in the ketogenic diet alone group in the SN was a tendency to increase DA 2 weeks after brain surgery (5 weeks in diet total) (*p* = 0.09) and decreased turnover rate after 4 days and 2 weeks (*p* = 0.026, *p* = 0.056, respectively) followed by a tendency to increase turnover 4 weeks after the operation (*p* = 0.07). These fluctuations corresponded with rat locomotor activity. Therefore, the ketogenic diet itself might, to some extent, influence DA metabolism. 6-OHDA lesion caused a progressive decrease of DA concentration in both structures, with the first significant decline already visible 4 days post-operation in the STR and after 2 weeks in the SN. DA turnover rate in the SN decreased while in the STR it was was enhanced due to the nigrostriatal system lesion.

Serotonin level and its metabolism did not show any major changes in the tissue. The only increased turnover rate in the SN was observed 4 days after surgery in ketogenic diet alone and with 6-OHDA groups (5-HIAA/5-HT, *p* = 0.048 and *p* = 0.0215, respectively, Supplementary Table S2).

**Figure 5:**
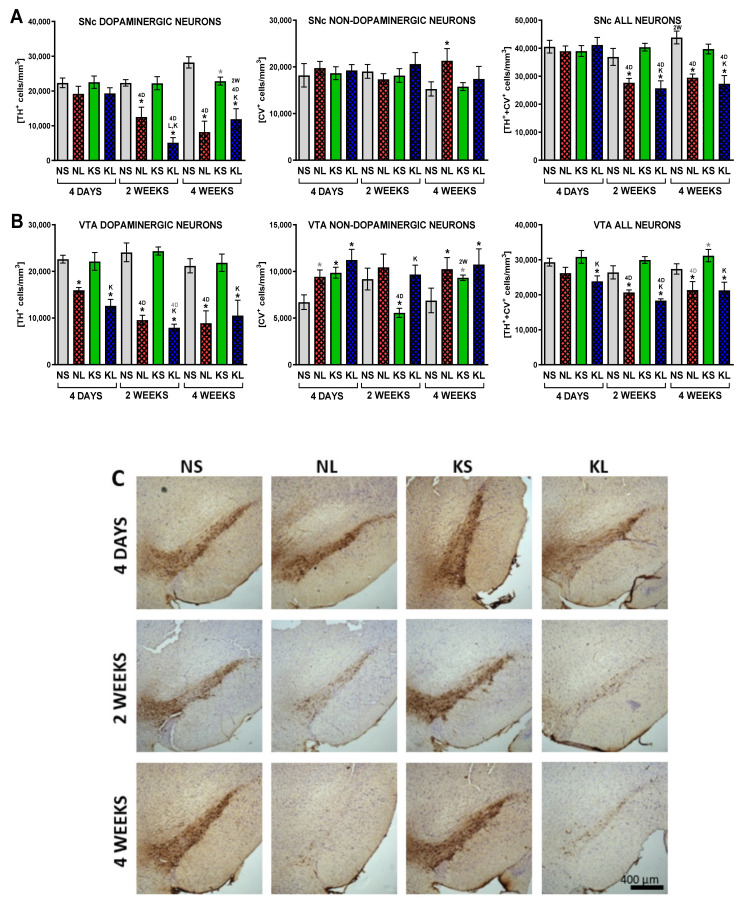
Stereological counting of dopaminergic and non-dopaminergic neuron density in the SN (**A**) and VTA (**B**) with representative photographs (**C**) stained for TH and cresyl violet. Data are presented as mean ± SEM. Analysis was performed at time-points 4 days, 2 weeks, and 4 weeks after brain surgery. Animals were kept on ketogenic diet for 3 weeks in advance. *p* value ≤ 0.05 was set as significance threshold and marked as * vs. normal diet + sham (NS group), L vs. normal diet + 6-OHDA lesion (NL group), K vs. ketogenic diet + sham (KS group), KL stands for ketogenic diet + 6-OHDA lesion group, 2W vs. 2 week time-point, 4D vs. 4 day time-point. Statistical trends 0.05 < *p* ≤ 0.1 were marked in grey symbols. Statistics: 3 way ANOVA with LSD post hoc test. Number of animals was 4–7 per group.

Finally, stereological analysis of neurons confirmed the progressing decline in the density of dopaminergic (TH+) neurons in the SNc (remaining 86, 56, 29% neurons of respective control in sequencing time-points) due to the 6-OHDA injection (Figure 5). A significant loss of dopaminergic neurons was documented 2 weeks after lesioning in the SNc and after 4 days in the VTA. The loss of dopaminergic phenotype preceding neuron death (a decrease in TH+/CV+ neurons while an increase in TH-/CV+ cells) was observed in the VTA but not SNc. Ketogenic diet had no protective effect on dopaminergic neurons challenged with 6-OHDA toxin.

Expression of TH in the STR analyzed densitometrically also did not show any protective effect of the ketogenic diet on the 6-OHDA induced lesion of the dopaminergic system (Figure S1a). The same effect was visible in the TH protein amount analyzed by Western blot in the SN (Figure S1b).

### 2.3. In the Brain, Glycogen Energy Reserve Does Not Change, but 7 Weeks of Ketogenic Diet Increases It in the Liver

**Figure 6:**
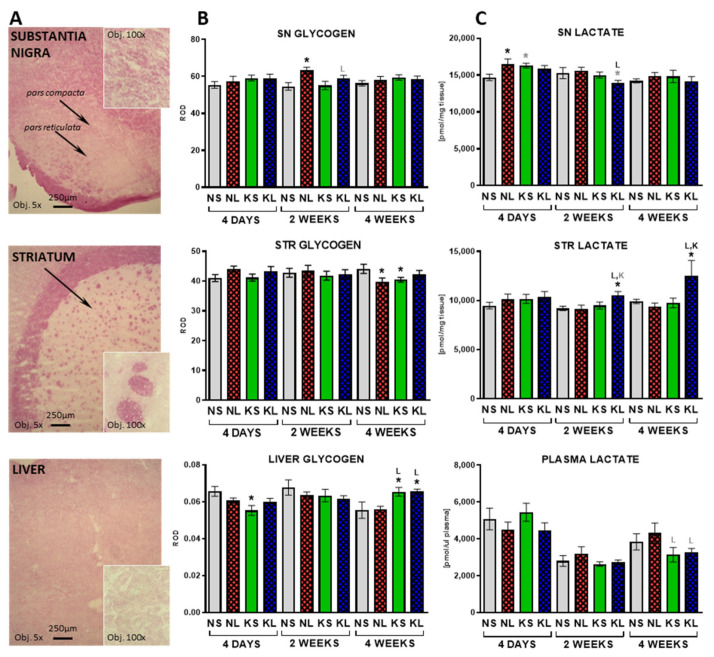
Glycogen was measured on stained tissue sections from SN and STR and liver (**A**) as Relative Optical Density (ROD) (**B**). Lactate (**C**) was measured in plasma and brain homogenates from dopaminergic structures SN and STR. Data are presented as mean ± SEM. Analysis was performed at time-points 4 days, 2 weeks, and 4 weeks after brain surgery. Animals were kept on ketogenic diet for 3 weeks in advance. *p* value ≤ 0.05 was set as significance threshold and marked as * vs. normal diet + sham (NS group), L vs. normal diet + 6-OHDA lesion (NL group), K vs. ketogenic diet + sham (KS group), KL stands for ketogenic diet + 6-OHDA lesion group. Statistical trends 0.05 < *p* ≤ 0.1 were marked in grey symbols. Statistics: 3-way ANOVA with LSD post hoc test. Number of animals was 5–7 per group.

Glycogen energy reserves are strictly correlated with glucose availability. Staining and densitometry analysis (Figure 6) determined its amount in the brain and liver tissues. At 4 days after operation, the only significant change of glycogen staining was a decrease observed in the liver in the ketogenic control group (KS). Dopaminergic lesion increased glycogen levels in the SN after 2 weeks (*p* = 0.003). At 4 weeks after brain operation (seventh week on the diet), liver glycogen levels significantly increased in both ketogenic groups (KS *p* = 0.017 and KL *p* = 0.017) to the same extent. At the same time, small decreases were observed in the STR in the lesion-only (NL) and diet-only (KS) groups.

Lactate is produced in the brain by astrocytes and shuttled to neurons upon higher energy demand. In this study, lactate levels were increased in the SN at the fourth day after operation in the lesioned group (NL) and tended to increase in the ketogenic diet control group (KS) (Figure 6). Interestingly, lesioned animals fed a ketogenic diet (KL) for 5 weeks (2 weeks post-operation) showed an increased level of lactate in the STR but a slightly decreased level in the SN. Increased level of lactate in the KL group was still observed in the STR at the latest studied time-point, 4 weeks after the operation.

### 2.4. Mitochondrial Cx II Activity Significantly Decreases in the Liver after Prolonged Ketogenic Diet

**Figure 7:**
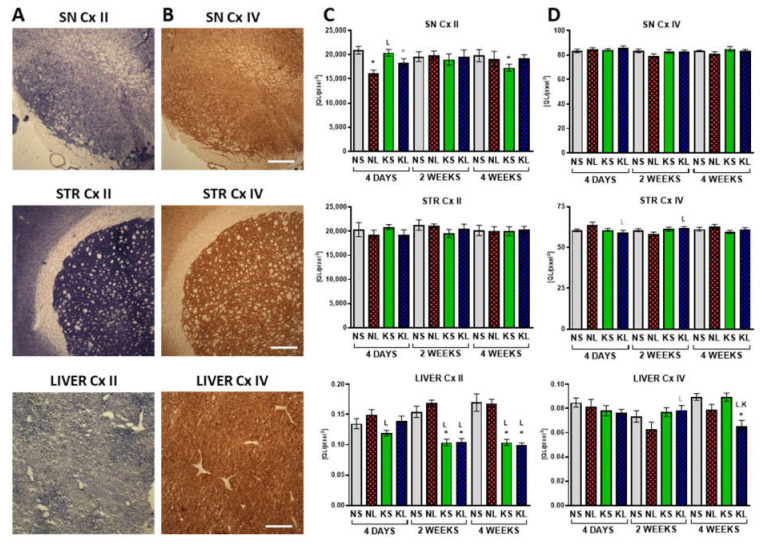
Cx II (**A**,**C**) and Cx IV (**B**,**D**) staining in brain and liver, representative photographs (**A**,**B**) and quantification (**C**,**D**). Staining was performed on tissue sections from SN and STR and liver as Relative Optical Density (ROD). Data are presented as mean ± SEM. Analysis was performed at time-points 4 days, 2 weeks, and 4 weeks after brain surgery. Animals were kept on ketogenic diet for 3 weeks in advance. *p* value ≤ 0.05 was set as significance threshold and marked as * vs. normal diet + sham (NS group), L vs. normal diet + 6-OHDA lesion (NL group), K vs. ketogenic diet + sham (KS group), KL stands for ketogenic diet + 6-OHDA lesion group. Statistical trends 0.05 < *p* ≤ 0.1 were marked in grey symbols. Statistics: 3-way ANOVA with LSD post hoc test. Number of animals was 5–7 per group. Scale bar represents 400 µm.

Cx II and Cx IV mitochondrial activities were assessed on tissue sections from the brain and liver (Figure 7) to check the oxidative phosphorylation (OxPhos) functioning. The only significant change in the Cx IV activity was a decrease observed in the liver 4 weeks after operation in the lesioned group (KL, *p* < 0.001, fed with a ketogenic diet for 7 weeks in total). Cx II is also a functional link between the TCA cycle and OxPhos. Cx II activity decreased in the SN due to the lesion (NL, *p* < 0.001 and KL, *p* = 0.065, 4 days). At later time-points only a decrease in the control ketogenic group was documented in the SN at 4 weeks post-lesion (KS, *p* = 0.015). Interestingly, in the liver, the ketogenic diet significantly decreased Cx II activity. At 4 days, this effect was counteracted by a non-significant increase due to the lesion, but at the later time-points (2 and 4 weeks post-operation = 5 and 7 weeks on the diet, respectively), both ketogenic groups (KS and KL *p* < 0.001) showed strong decreases of the same extent.

### 2.5. Gluconeogenesis in the Liver during Ketogenic Diet

**Figure 8:**
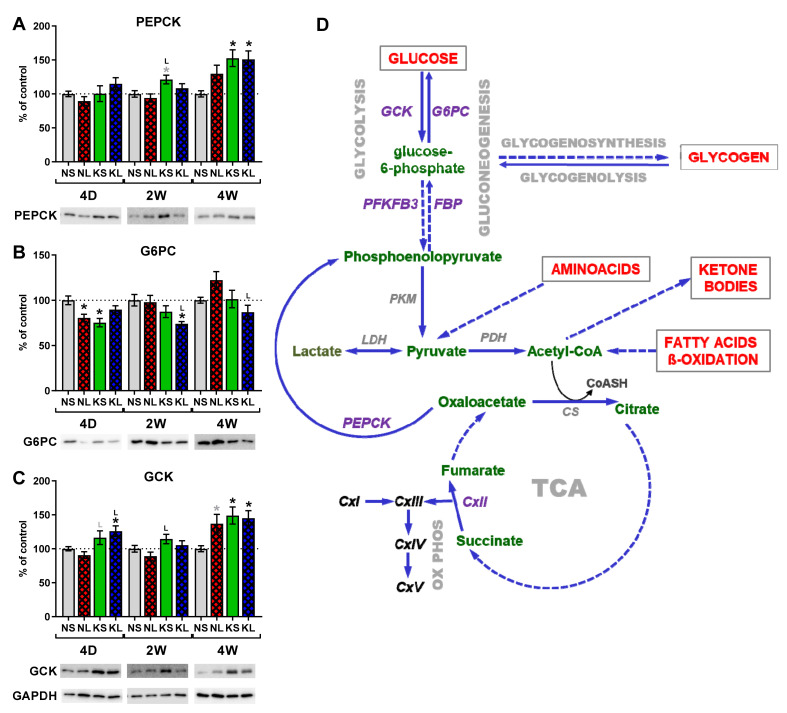
Protein expression in liver, (**A**–**C**) Western blot and schematics of liver energy metabolism (**D**). Analysis was performed on liver homogenates in triplicate for each animal, from *n* = 6 animals per group. Data are presented in % of control as mean ± SEM. Analysis was performed at time-points 4 days, 2 weeks, and 4 weeks after brain surgery. Animals were kept on ketogenic diet for 3 weeks in advance. *p* value ≤ 0.05 was set as significance threshold and marked as * vs. normal diet + sham (NS group), L vs. normal diet + 6-OHDA lesion (NL group), K vs. ketogenic diet + sham (KS group), KL stands for ketogenic diet + 6-OHDA lesion group. Statistical trends 0.05 < *p* ≤ 0.1 were marked in grey symbols. Statistics: 3-way ANOVA with LSD post hoc test. (**C**) Liver energy metabolism. Full arrows indicate direct processes, while dashed arrows indirect, with additional processes in-between. CS—citrate synthase; FBP—fructose 1,6-bisphosphatase; G6PC—glucose-6-phosphatase; GCK—glucokinase; LDH—lactate dehydrogenase; OxPhos—oxidative phosphorylation; PDH—pyruvate dehydrogenase; PEPCK—phosphoenolpyruvate carboxykinase; PFKFB3—6-phosphofructo-2-kinase/fructose-2,6-biphosphatase 3; PKM—pyruvate kinase; TCA—tricarboxylic acid cycle.

To check if maintained normoglycemia in the plasma, despite the ketogenic diet, was due to the increased gluconeogenesis in the liver, we studied protein expression of key enzymes in the gluconeogenic pathway (Figure 8). Phosphoenolpyruvate carboxykinase (PEPCK, also known as PCK or PCK1) and glucose-6-phosphatase (G6PC) catalyze the irreversible reactions in gluconeogenesis [40]. On the other hand, glucokinase (GCK) catalyzes the process of glucose metabolism, exactly opposite to that of G6PC in gluconeogenesis [41].

PEPCK catalyzes the decarboxylation of oxaloacetic acid to phosphoenolpyruvate and stimulates hepatic glucose production. The lack of posttranslational modifications influencing its activity makes its protein level a good marker [41]. We observed increased PEPCK expression in the liver after the ketogenic diet starting from 2 weeks post-operation (KS, fifth week on the diet). After 4 weeks, both ketogenic diet treated groups (KS and KL) showed significant increases.

G6PC hydrolyses glucose-6-phosphate to glucose in the final step of gluconeogenesis therefore directly contributes to the blood glucose level. We observed decreased expression of G6PC 4 days post-operation in KS and NL groups. Later, its amount increased in time to reach normal control levels after 4 weeks. Another significant decrease of G6PC protein was visible after 2 weeks in lesioned animals on the ketogenic diet (KL).

GCK (also known as hexokinase IV) catalyzes the first step in glucose metabolism. By its phosphorylation, glucose is trapped inside the cell. Therefore, GCK functions as a glucose sensor. Its expression in the liver increased after 4 weeks in both groups treated with the ketogenic diet (KS and KL) as well as in the lesioned only animals. KL group also showed increased GCK expression after 4 days.

## 3. Discussion

### 3.1. Proof of Induction of Hyperketonaemia

In order to induce stable, physiological ketosis, animals were fed with the ketogenic diet for 3 weeks prior to lesioning. The diet was stringent and contained only 1% of carbohydrates, decreased amount of protein (8%), and 70% of fat. Animals were maintained for further 4 weeks of such diet after operation. No other food was administered; even feces were regularly removed from the cage to prevent coprophagy. As expected, rats on a ketogenic diet gained weight slower than controls (Figure 1). In total, animals were kept for 7 weeks on the ketogenic diet. No previous study analyzed the effect of such a prolonged ketogenic diet on the PD animal model. Previous studies showed that the normal rat blood concentration of ketone bodies in physiological conditions is very low (<0.22 mM) but increases four fold (up to 1.15 mM) after 3 days on a ketogenic diet [42]. To verify the induction of physiological ketosis, we analyzed the levels of ketone bodies in the form of b-HB both in blood and brain dopaminergic structures (Figure 2). B-HB increase in plasma and in the brain was very large, proving long-term ketosis. The highest b-HB levels were at the first week after operation, probably due to the rats’ convalescence, and stabilized over the next weeks. Therefore, the diet was effective in the induction of physiological ketosis in animals.

### 3.2. Lack of Neuroprotection

In this study, a low dose of 6-OHDA toxin (3 µg) was injected into the MFB fibers, passing from the SN to the STR, causing progressive, medium size degeneration of the dopaminergic neurons, similar as in our previous studies [35,36,39]. Such progression of degeneration from terminals towards the cell body and size of the lesion still allows for functional compensation of the deficits and for neuroprotection. Despite counting both dopaminergic and non-dopaminergic neurons in the SN pars compacta and VTA at three time-points of treatment, no signs of neuroprotection were detected.

Among the actually documented in vivo neuroprotective effects of the ketogenic diet in the SN, only two previous studies reported fewer dopaminergic neuron deaths due to the 6-OHDA or MPTP [22,23]. Another study proving neuroprotection but after external b-HB infusion in osmotic pumps instead of the ketogenic diet was presented by Tieu et al. [21]. Other studies showed potential neuroprotection but not directly. Shaafi [24] reported an improvement in motor dysfunction after the ketogenic diet but did not document DA neuron counts; therefore, the observed improvement might have been functional rather than structural. The improvement could be due to the better compensation, not actual neuroprotection, given the large dose of 6-OHDA injected directly into the SN, causing most probably a near-complete loss of neurons as shown in previous studies [43,44]. Similarly, Joniec-Maciejak [45] reported an improvement in DA and metabolite levels due to the octanoic acid administration but did not document cell counts.

In the previous studies where neuron loss was induced by MPTP, the neuroprotective effect could be partially due to the inhibition of microglia activation and anti-inflammatory effect of ketone bodies [23]. Anti-inflammatory b-HB effects were reported as inhibition of microglia activation [23] and direct inhibition of NLRP3 inflammasome [46] (for review, see also [47]). Multiple studies have shown that microglia activation is an important factor in MPTP-induced cell death. Thus, inhibition of microglia activation acts as a neuroprotective in the MPTP-induced PD animal model [48,49,50]. In contrast, in the 6-OHDA model used here, microglia activation is either very small or mostly not detectable [35,36]. In our model where 6-OHDA is injected into the MFB, there is no microglia activation in the SN, as we showed before [35,36,39].

As an opposite to 6-OHDA, MPTP also acts via astrocytes which metabolize this toxin to the active form MPP+. Astrocytes have been shown to act protective towards neurons, among many functions by ketone body production [51,52,53]. Astrocytes also benefit from ketone body substitution from the periphery more than neurons due to the greater b-HB uptake [11,54]. Astrocyte dysfunction affects neuron viability [35,36]. Ketone bodies provided by the diet or applied directly might have relieved the burden put on astrocytes by the toxin, and in this way may also cause neuroprotection in MPTP/MPP+ models [54].

Cheng et al. [22] showed neuroprotection in the 6-OHDA model. They injected 20 µg of 6-OHDA into the STR terminals. Such treatment normally induces mostly loss of dopaminergic terminals, which tend to partially regrow with time. Such lesion technique, as a consequence, causes slow, progressive retrograde neuron degeneration, causing very small lesions in the SN dopaminergic cell bodies [55]. Surprisingly, they showed an approximate 45% decrease in TH+ neuron count in the SN and approximate 50% decrease of TH in the STR. In our previous studies, 15 µg 6-OHDA injection into the STR caused only a 29% loss of TH+ neurons in the SN 2 weeks after lesion, but after 4 weeks, this decrease was only 20% [55]. Such effect is often observed as DA neurons first lose their TH expression and only after a longer time either die or recover [35,38,56]. That is why both TH and Nissl staining should be performed on the same tissue sections. The effect that Cheng et al. observed could be due to the improved regeneration of DA neurons thanks to the ketogenic treatment, not necessarily neuroprotection per se [22], which would also be a very interesting observation if documented.

Despite three time-point timeline analyses of behavior and neuron count as well as DA level and metabolism, our study did not show any signs of neuroprotection due to the long-term rigorous ketogenic diet in a 6-OHDA-induced medium size lesion animal model of early PD. In conclusion, toxin-based animal models such as 6-OHDA are designed to study specific mechanisms of neuroprotection, here antioxidant processes. These models usually use fast-acting oxidative stress inducers, so clearly, antioxidants should be used for neuroprotection against those toxins. Even though the ketogenic diet was shown previously to have antioxidant properties, here it did not act neuroprotective in a model of retrograde degeneration of dopaminergic cells. The pathogenic cause and exact mechanisms of neuronal death in PD are still unknown, and so are the targets for neuroprotection.

### 3.3. The Effect on Behaviour and DA Metabolism in the STR

In our study, control rats fed a ketogenic diet showed temporarily increased locomotor activity, corresponding to the increased DA level and decreased turnover in the SN. The exact explanation, besides food-seeking behavior, is so far unavailable. Other studies showed variable effects of ketogenic diet treatment itself, from greater locomotor activity [11] to significantly decreased locomotor activity [57]. Moreover, previous studies showed that the ketones or ketogenic diet itself did not change DA level or turnover ratio in the STR or SN of animals [21,22,58]. Church et al. [58] showed significantly increased turnover but only in the cortex. In the midbrain, the DA turnover was elevated but statistically not significant.

A medium-sized dopaminergic lesion has a natural tendency to functionally compensate for the loss of neurons, as seen in the early PD stage. Therefore, similar to the previous studies [35], after an initially decreased locomotor activity, spontaneous return to the near-normal levels of activity was observed in lesioned rats. Lesioned animals on a ketogenic diet showed a tendency to increased locomotion after 4 weeks post-operation as compared to the lesioned animals on a regular diet. Previous studies describing ketogenic diet neuroprotection in PD models all showed partial reversal of motor dysfunction [21,23,24] between 1 and 2 weeks post-lesioning. Similarly, reversal of DA and DOPAC depletion due to the neuroprotective effect of the ketogenic diet were reported in two studies [21,22].

Early in PD, an increase in DA turnover has been hypothesized to occur as one of the compensatory mechanisms against partial dopaminergic neuronal loss. It was validated in a nonhuman primate PD model, where DA turnover increased before the parkinsonian symptoms [59,60]. Similarly, in our current and previous studies [35,39], DA turnover increased significantly in the STR due to the medium lesion in order to maintain higher DA levels (Figure 4). Interestingly, 4 weeks after operation, lesioned animals fed with a ketogenic diet showed a tendency to normalize DA level and turnover in the STR.

Although our study effect of increased locomotor activity and normalized DA turnover was small and only detected after 4 weeks, this finding goes along with the hypothesis that increased energy requirements, necessary for functional compensation of partial degeneration of dopaminergic neurons, could be supported by ketone bodies supplied by a ketogenic diet [35,36,37]. Nevertheless, at the earliest studied time-points (3 and 5 days) when locomotor disability was still visible prior to compensation, the ketogenic diet even aggravated this deficiency. Therefore, this study indicates that maybe the ketogenic diet could be therapeutically helpful to support late functional compensatory mechanisms but not necessarily act against the neurodegeneration itself.

### 3.4. The Effect on Glucose

In this study, despite a long-term ketogenic diet containing only 1% carbohydrates, blood glucose remained at the control level. In some previous studies on ketogenic diets, glucose levels have been reported to be decreased [57,61] but mostly unchanged in the plasma of adult rodents [42,53,62]. Some explanation of such effect was published by Leino et al. [42], who performed timeline studies and showed an initial decrease in blood glucose followed by a return to normal levels by 21 days. Our analysis was started after 3 weeks of diet; therefore, animals have probably already adjusted their metabolism to maintain normoglycemia. Interestingly, many previous studies on the ketogenic diet in rodents showed impairments in glucose tolerance and insulin sensitivity, and this was attributed to a possible adaptation to maintain blood glucose levels against insufficient amounts of carbohydrates (see [63]). Importantly, none of the studies reporting neuroprotection in animal PD models actually measured blood glucose so far [21,22,23,24]. It is possible that besides increased ketone bodies, the long-term glucose decrease is required for the neuroprotective effect of the ketogenic diet.

### 3.5. Length of the Diet

The length of the ketogenic treatment is also a very important factor. As mentioned above, short-term treatments induce other effects than longer ones. Włodarek [12] reported that on a diet with very low carbohydrate content, glycemia, although decreasing, remains at its physiological level. In these conditions, the glucose is formed de novo from two sources: glucogenic amino acids and glycerol, released from triglycerides. In the first days of the ketogenic diet, the main source of glucose is via gluconeogenesis from amino acids, while in the days following, the importance of glycerol as a glucose source increases [17,64]. We chose to study the stable, long-term diet effects, starting after 3 weeks with 4 further weeks after lesion. The clearest and most significant changes compared to normal diet were observed mostly after the longest time: 7 weeks on the diet. Previous studies reporting neuroprotection in animal PD models analyzed effects of shorter periods on the diet—2 weeks prior and 2 weeks after lesion [22], 11 days prior and 2 weeks after lesion [24], or 1 week prior and 1 week after lesion [23]. Recently, Arsyad et al. [65] showed that a 60-day, long-term ketogenic diet consisting of 5.66% of carbohydrate, 86.19% fat, and 8.15% protein induced metabolic acidosis, anemia, and reduced antioxidant enzyme level in healthy Wistar rats. In contrast to our study, they observed significantly reduced glucose levels in the blood. Previous studies in mice reported hepatic inflammation and lipid accumulation concomitant with the development of NAFLD (non-alcoholic fatty liver disease) and associated hepatic insulin resistance (see [63]). Therefore, the long-term ketogenic diet itself carries serious side effects, and such treatment is not as healthful as generally expected. Therefore, it is important to study the long-term effect of such treatments.

### 3.6. The Effect of Age

The age of animals is an important issue in the aspect of ketogenic diet effectiveness. Used in this study, young-adult rats have major metabolic flexibility. Hernandez et al. [61] showed that in young rats with a ketogenic diet, glucose levels were higher than in aged rats while they adapted to the ketogenic diet, up to 30 days. It was shown before that induction of hyperketonemia decreased glucose metabolism in 20-day-old rats, but did not alter glucose metabolism in adult rats [11]. Additionally, cerebral uptake of b-HB is higher in young than adult rats [9,10,11,27,28]. There is an inverse relationship between age and the brain’s capacity for ketone metabolism. Similarly, the highest effectiveness of the ketogenic diet in epilepsy treatment was documented in children. Since PD is strongly related to aging, the question arises if the aging human brain is able to utilize ketone bodies at therapeutically relevant level. For review, see [9,10,11].

### 3.7. Liver—Glycogenolysis and Gluconeogenesis as Possible Sources of Glucose

The liver plays a major role in the maintenance of glucose homeostasis by regulation of the glucose storage (via glycogenesis) and de novo glucose production and release (via glycogenolysis and gluconeogenesis) [66,67,68].

Due to the diminished dietary availability of glucose and, on the other hand, maintained normoglycemia in blood in our study, it would be expected that glycogen degradation was enhanced as described in other studies [67,69]. However, we did not note overall decreased glycogen levels due to the ketogenic diet, neither in the brain nor liver, probably because the first tested time-point was after more than 3 weeks in the diet. Surprisingly, we even observed increased glycogen amount in the liver after 7 weeks on the ketogenic diet.

Gluconeogenesis is a multistep metabolic process that generates glucose from pyruvate or a related three-carbon compounds (lactate, alanine) [52]. During prolonged fasting, hepatic gluconeogenesis is increased [67] along with the expression of the involved enzymes, especially PEPCK and G6PC [41,57,70,71].

PEPCK transforms oxaloacetate into phosphoenolpyruvate and is one of the earliest, rate-limiting steps in gluconeogenesis. Additionally, in our studies, PEPCK protein expression increased significantly due to 7 weeks of the ketogenic diet, at least partially explaining the normoglycemia after such a stringent and long diet. Another study by Park et al. [69] showed similarly to our data that the increased hepatic glucose output in the ketogenic diet was associated with increased PEPCK expression. Along with this data, transgenic mice overexpressing PEPCK also exhibited an increase in hepatic glucose production [72]. However, conversely, Ribeiro et al. [41] showed a significant decrease in liver PEPCK and also no decreased glucose in rats fed the ketogenic diet for 6 weeks. Additionally, Hutfles et al. [73] as well as Bielohuby et al. [57] reported decreased levels of PEPCK in ketogenic diet-fed animals. Despite that, PEPCK protein expression directly relates to its activity, and our and recent results indicate that the changes in liver PEPCK are not necessarily accompanied by equivalent changes in gluconeogenesis [41].

Another key enzyme in gluconeogenesis, G6PC, directly catalyzing glucose production from glucose-6-phosphate (G6P), was not increased in or study. The regulation of G6PC has an added level of complexity because its activity depends on three transporters and a stabilizing protein [40]. Therefore, G6PC protein amount might also not directly correlate with its activity and actual glucose production. In the previous studies, protein and mRNA of G6PC mRNA significantly decreased instead of rising after 4 weeks of a stringent ketogenic diet, maybe due to decreased glucose in the blood [57]. Additionally, Bosma et al. [74] showed a lack of decrease in blood glucose level associated with ketogenic diet and reduction in G6pc2 gene expression, although in the pancreas. Interestingly, G6pc2 was inhibited as an acute effect after 24 hr fasting and strongly induced by high-fat feeding. These observations suggest that other unknown factors associated with the ketogenic diet modulate glucose levels.

The third enzyme studied: GCK acts directly opposite to G6PC and phosphorylates glucose to G6P, keeping glucose inside the cell and is a rate-limiting step for glycolysis in hepatocytes. It also links glucose influx with glycolysis and glycogen synthesis and is a highly regulated process [75]. Surprisingly, in our study, its protein expression in the liver increased significantly in both groups fed a ketogenic diet for 7 weeks.

Although we detected significantly increased PEPC protein expression, suggesting enhanced gluconeogenesis which could partially explain normoglycemia in a ketogenic diet, G6PC expression was not increased, and GCK enzyme was increased instead of lowered. Therefore, we cannot conclude that liver gluconeogenesis was responsible for normoglycemia in these ketogenic-treated animals. Metabolic pathways sparing available glucose besides gluconeogenesis and glycogenolysis must have also been involved. Possibly, the insulin regulation feedback could play a role. Dynamic metabolic network changes and more detailed metabolic studies are required to further explain this effect.

### 3.8. Mitochondria and OxPhos Cxs

Experimental in vivo data on the ketogenic diet’s influence on the mitochondrial respiratory Cxs activities and expression in either in the brain or liver are scarce. Fatty acid β-oxidation generates large amounts of NADH and FADH_2_, which donate electrons to Cx I and Cx II, respectively [73]. Since the ketogenic diet strongly rearranges energy metabolism and shifts the use of particular energy substrates, it would be of interest if ketogenic treatment influences oxidative phosphorylation.

In the brain, external administration of b-OHB, without the ketogenic diet, reduced production of ROS, and b-HB supplementation was not neuroprotective against MPTP when Cx II activity was diminished [21]. Therefore, it was suggested that Cx II was mediating this process by bypassing the Cx I deficit caused by the MPTP toxin and increasing overall mitochondrial respiration. Despite such promising effects of b-HB itself, in our studies on the ketogenic diet, no major changes were observed in either Cx II nor IV in the brain dopaminergic structures SN or STR. No other studies are available on this subject from in vivo PD model studies so far.

On the other hand, interesting changes were observed in the liver both after 2 and 4 weeks post-lesion (5 and 7 weeks on ketogenic diet, respectively). We observed a strong decrease in Cx II activity staining in both groups treated with the diet (KS and KL). Surprisingly, the only other study on Cx II in the liver after 4 weeks of the ketogenic diet by Hutfles et al. [73] showed no significant changes in the protein level of Cx II subunit A, while Cx IV subunits were decreased. Garnol et al. [76] stipulated that animals fed 6 weeks with a high-fat diet (71% fat, 18% proteins, 11% carbohydrates) had disturbed Cx II activity. Additionally, Grattagliano et al. [77] showed a decreased amount of Cx II subunit 30 kDa in animal livers after 2 and 8 weeks on a high-fat diet (71% fat, 11% carbohydrate, 18% protein). Decreased Cx II was also detected after liver ischemia [78], and Cx II flux was reduced severely in the presence of fatty acids in the livers of animals who developed type 2 diabetes [79]. Therefore, as we observed, decrease in Cx II liver staining most probably is due to the long-term high-fat content in the diet, further suggesting that a chronic ketogenic diet is not easily tolerated by the liver and could have harmful side-effects.

### 3.9. Protein Amount in the Diet

A ketogenic diet must be low in carbohydrates, high in fat, but also low in protein contents to be clearly ketogenic [57]. The diet used in this study contained a low amount of protein (8%), only 1% of carbohydrates, and 70% of fat; therefore, its consumption for up to 7 weeks should have induced ketosis, which was proven by increased b-HB levels in blood and brain. Similar to our data, Oke et al. [80] reported that animals exposed to a low-protein diet (8%) in prenatal life and postnatally fed with a normal diet showed decreased Cx II amount in the liver. In the study by Bielohuby et al. [57], the increase in b-HB levels in plasma negatively correlated with the amount of protein in the diet. The high-fat/low-protein diet (75% fat and 10% protein) showed threefold higher concentrations of b-HB in serum as compared with controls and the high-fat/normal protein (55% fat and 30% of protein), which did not induce a significant increase in serum b-HB levels.

## 4. Materials and Methods

### 4.1. Animals and Brain Surgery

The experiments were carried out in compliance with the Animal Experiments Bill of January 21, 2005 (published in Journal of Laws no. 33/2005 item 289, Poland), and according to the NIH Guide for the Care and Use of Laboratory Animals. We also received approval from the Local Ethical Committee (947/2012; 1178/2015). All efforts were made to minimize the number of animals and their suffering.

Male Wistar HAN rats (Charles River, Sulzfeld, Germany), 3 months old at the beginning of the experiments, were kept at 12 h dark/light cycle (light from 06:00 to 18:00), 4–5 per cage, with free access to water and food. Control animals received normal diet (55% carbohydrates, 19% protein, 5% fat, fiber and ash as filling, calculated as weight %) while treated animals received ketogenic diet (1% carbohydrates, 8% protein, 70% fat, fiber and ash as filling, calculated as weight %) (Labofeed B standard and Labofeed B 70% fat, Wytwórnia Pasz Morawski, Kcynia, Poland, respectively). The main fat component was lard. The strict diet was kept for 3 weeks before the brain operations and maintained until the end of experiment. Body weight was monitored. Animal feces were systematically removed to avoid unwanted carbohydrate contamination due to coprophagy.

Stereotaxic brain operations were performed under ketamine and xylazine anesthesia (Biowet, Puławy, Poland) as published earlier [39]. Desipramine (15 mg/kg *ip*, Sigma-Aldrich, Darmstadt, Germany) was administered 30 min before lesioning to protect the noradrenergic terminals. The animals were bilaterally injected with 6-OHDA HBr (3 μg base/3 μL per side), dissolved in a 0.2% ascorbic acid (both from Sigma-Aldrich, Darmstadt, Germany) into the passing fibers of medial forebrain bundle (MFB), at coordinates: AP: 1.4 mm, L: ±1.6 mm, V: 8.7 mm from bregma [81]. Control—sham-operated rats received solvent in the same way.

### 4.2. Behavioural Analysis Using Automated Actometers

Rat locomotor activity (path length, locomotion, and resting times) and rearings (total, free and supported number, duration) were measured at four time-points after operation (3, 5, 13, 27 days) using computerized actometers (ACTIFRAME-SYSTEM, GERB Elektronik GmbH, Berlin; Germany with ARNO software) as described before in detail by [35,82]. Animals were placed individually in cages with free access to food and water and analyzed for 18 h, consisting of both light and dark phases of the day. Each analysis session included animals from all treatment groups from the same post-operation day. If the same animal was tested twice, the analyses were at least 10 days apart.

### 4.3. HPLC-EC Analysis of DA, Its Metabolites, and Turnover Rates

Rats were decapitated at fourth, fourteenth, or twenty-eighth day after operation. Left STR and SN were immediately dissected and frozen on dry ice. The samples were weighed, homogenized, and deproteinized in 0.1 M perchloric acid. The levels of DA and its metabolites: 3,4-dihydroxyphenylacetic acid (DOPAC), 3-methoxytyramine (3-MT), homovanillic acid (HVA), as well as noradrenaline, serotonin (5-HT), and its metabolite, 5-hydroxyindoleacetic acid (5-HIAA), were assessed using HPLC method with electrochemical detection, as published previously [55]. Samples were injected into the HPLC system (32 °C, column Hypersil Gold C18, 100 × 3.0 mm, 3 μm, (Thermo Scientific, Waltham, MA, USA) and analytic cell 5010 Coulochem III, (ESA, Inc., Chelmsford, MA, USA)). The mobile phase was composed of 50 mM NaH_2_PO_4_ x 2H_2_O; 40 mM citric acid; 0.25 mM 1-octanesulfonic acid sodium salt; 0.25 mM EDTA; 1.3% acetonitrile; 2.4% methanol. The data were quantified using the area under the peaks and external standards with Chromeleon software (Dionex, Sunnyvale, CA, USA). The turnover was calculated as metabolite to neurotransmitter ratio.

### 4.4. Immunohistochemistry and Stereological Counting Neurons

Immunohistochemistry: After the decapitation, the right hemisphere of the brain was rapidly removed, post-fixed in cold 4% paraformaldehyde for 7 days, and cryoprotected in 20% sucrose solution in phosphate-buffered saline (PBS). The brains were then cut on a freezing microtome into 25 μm frontal sections (AP –4.4 to 6.6 mm and AP = +2.2 to −1.5 mm from Bregma for SN pars compacta (SNc)—ventral tegmental area (VTA) and STR, respectively, according to Paxinos and Watson (2007)) by the principles of the stereological rules and stained as described before [56]. Free-floating sections were incubated for 48 h at 4 °C in primary antibodies (anti–tyrosine hydroxylase (TH), 1: 2’500) and in secondary antibodies (anti-mouse biotinylated, 1:200, Vector Laboratories, UK) for 30 min, processed using an ABC-peroxidase kit (Vector Laboratories, Burlingame, CA, USA), and 3,3’-diaminobenzidine as a chromogen. Subsequently, the sections containing SNc—VTA stained for TH were counterstained with 1% cresyl violet (CV) with Nissl method. All sections were cover-slipped in a Permount medium (Fisher Scientific, Waltham, MA, USA).

Stereology: Neurons stained with TH^+^/CV^+^ and TH^-^/CV^+^ were counted stereologically in the SNc and VTA as described previously [83]. The sampling of tissue was started at random from the beginning of the structure, systematic and uniform—every sixth section was taken. At least 8 sections through the entire length of the SNc and VTA, respectively, were sampled.

Stereological counting was performed using a light microscope (Leica, Denmark) equipped with a projecting camera (Basler Vision Technologies, Ahrensburg, Germany) and an automated stage (PRIOR ProScan) controlled by a newCAST (Visiopharm, Hørsholm, Denmark) software. The analyzed regions were outlined under lower magnification (5x), and their areas were estimated. The number of stained cells was calculated under 63x magnification using a randomized meander sampling and the optical dissector methods. The dissector’s height was 10 μm. The area of counting frame was 8302.8 μm^2^ and covered 30% of the screen area. Meander sampling probed 15% of the delineated regions of interest, resulting in counting not less than 150 cells from each animal.

Densitometry: The intensity of neuronal terminal TH staining in the STR was estimated by high-precision scans of sections (Scanner Epson Perfection V750 Pro, Seiko Epson Corporation, Nagano, Japan) using Multi Gauge software (Fujifilm Holdings Corporation, Tokyo, Japan). Regions of interest were outlined, and mean quant level per area (QL/pixel^2^) was quantified from 10–12 sections per animal [56].

### 4.5. Quantitative Assays for Energy Metabolism Substrates

Tissue samples were homogenized in 0.1 M perchloric acid, and pH of supernatants was adjusted with KOH. The plasma was prepared by centrifugation. The assays for lactate and b-HB were performed on both tissue homogenates and plasma, using fluorescence PicoProbe kits, according to the manufacturer protocols (K638-100, K651-100, BioVision, Milpitas, CA, USA). Glucose assay on plasma samples was performed according to the manufacturer protocol (BIOLABO, Maizy, France). For signal detection on 96-well plates, a Tecan 200 Infinite spectrophotometer (Tecan Group, Ltd., Männedorf, Switzerland) was used. Standard curves were prepared fresh on each well plate separately.

### 4.6. Mitochondrial Cx II and Cx IV Staining in the Tissue Sections

One brain hemisphere and liver were dissected immediately after decapitation and frozen in *n*-heptane at dry ice. A total of 10 μm tissue sections were stained as published before [37]: for total staining for Cx II (10 mM PBS, 1.5 mM nitrotetrazolium blue, 130 mM sodium succinate, 0.2 mM phenazine methosulfate, 1 mM sodium azide), or for non-specific staining (additionally with 130 mM malonate), and for Cx IV: total staining (50 mM disodium phosphate, 0.1 mM cytochrome c, 4 mM 3,3′-diaminobenzidine), or non-specific staining (additionally with 2 mM sodium azide). After high-precision scanning (Scanner Epson Perfection V750 Pro, Seiko Epson Corporation, Nagano, Japan), the region of interest was outlined and analyzed densitometrically (Multi Gauge software, Fujifilm Holdings Corporation, Tokyo, Japan). The results were presented as the mean of QL/pixel^2^ (Quantum Level).

### 4.7. Glycogen Detection in the Tissue Sections

Brain and liver 10 μm fresh frozen tissue sections were air-dried and incubated in 0.5% periodic acid. After washing in water, slides were incubated in Schiff reagent, washed again, dehydrated, and cover-slipped. Control sections were preincubated with 0.2% amylase at 37 °C for 30 min. Control staining was also performed with saturated solution of dimedone at 60 °C for 10 min. The intensity of glycogen staining was estimated on high-precision scans of tissue sections (Scanner Epson Perfection V750 Pro, Seiko Epson Corporation, Nagano, Japan) using Multi Gauge software (Fujifilm Holdings Corporation, Tokyo, Japan). Regions of interest were outlined, and mean quant level per area (QL/pixel^2^) was quantified from 2–3 sections per animal.

### 4.8. Western Blot

Frozen tissue samples were lysed in RIPA buffer (50 mM Tris; 150 mM NaCl; 1% NP-40; 0.1% SDS; 0.5% sodium deoxycholate; pH 7.4) supplemented with protease and phosphatase inhibitors as published before [35,36,37]. Protein concentration in supernatants was determined using Pierce^TM^ BCA Protein Assay Kit (Thermo Scientific, Waltham, MA, USA). Protein samples were denatured and resolved by SDS-PAGE. Transfer to 0.2 µm PVDF membranes (Roche Diagnostics, Indianapolis, IN, USA) was performed with semi-dry, discontinuous buffer system in Trans-Blot^®^ Turbo^TM^ (Bio-Rad, Hercules, CA, USA).

Blots were probed with primary antibodies against tyrosine hydroxylase (TH) (MAB5280, Merck KGaA, Darmstadt, Germany), phosphoenolpyruvate carboxykinase (PEPCK) (sc-271029), glucokinase (GCK) (sc-17819) (Santa Cruz Biotechnology, Dallas, TX, USA), glucose-6-phosphatase (G6PC) (ab83690) (Abcam, Cambridge, UK), and glyceraldehyde-3-phosphate dehydrogenase (GAPDH) (sc-166545) (Santa Cruz Biotechnology, Dallas, TX, USA). Secondary anti-mouse/goat (sc-2005) or anti-rabbit/goat (sc-2005) (Santa Cruz Biotechnology, Dallas, TX, USA) antibodies conjugated with horseradish peroxidase were used. Detection was performed using chemiluminescence solution (0.1 M Tris; 5.3 mM H_2_O_2_; 1.25 mM luminol; 2.0 M 4-iodophenylboronic acid) [84]. PEPC, GCK, and G6PC were normalized to GAPDH expression. Analysis was performed in triplicate for each animal, from *n* = 6 animals per group. After TH immunodetection, the membranes were stained with Coomassie blue, and total protein staining in a lane was used as a loading control for each sample as described in [85].

### 4.9. Statistical Analysis

Results are presented as mean ± standard errors of mean (SEM). The statistical analysis of results was performed using STATISTICA 10.0 software (StatSoft Inc., Tulsa, OK, USA). *p* ≤ 0.05 was considered as statistically significant, and 0.1 ≥ *p* ≥ 0.05 were considered as trends. Analyses were performed by 3-way factorial ANOVA with Fisher Least Significant Difference post hoc test or 2-way ANOVA with LSD post hoc test and *t*-test for comparison of groups in time. For motor behavior analysis, repeated measures test was used. All groups consisted of 5–8 animals, and for behavioral studies, 8–26 animals.

## 5. Conclusions

Our results do not confirm the neuroprotective effect of a ketogenic diet or hyperketonemia in the 6-OHDA-induced animal model of PD. Increased level of ketone bodies in the brain and plasma was not enough to provide neuroprotection in this PD model; therefore, additional processes must be involved. Probably, the decreased glucose level and/or tissue sensitivity to glucose or insulin is a more important factor than the ketone support itself. Our further hypothesis could be that ketogenic energy support could be relevant in diseases where astrocytic energy support is decreased towards the neurons. Further differences between human and animal responses to the ketogenic diet should be taken into consideration. Caution should be taken when applying a stringent long-term ketogenic diet to elderly PD patients as it could negatively impact liver functioning. A ketogenic diet, besides, epilepsy would probably be more effective in Alzheimer’s disease [86] and mitochondrial deficits diseases rather than in PD.

## Figures and Tables

**Table 1:** Analysis of spontaneous rat behavior. Data presented as path length; locomotion, resting, and rearing times; number of all rearings, side wall rearings (supported), and free rearings (unsupported) measured in actometers, showed as mean raw values ± SEM. Analysis was performed at time-points 3, 5, 13, 27 days after brain surgery. Animals were kept on ketogenic diet for 3 weeks in advance. *p* value ≤ 0.05 was set as significance threshold and marked as * vs. normal diet + sham (NS group), L vs. normal diet + 6-OHDA lesion (NL group), K vs. ketogenic diet + sham (KS group), KL stands for ketogenic diet + 6-OHDA lesion group. Repeated measure ANOVA with LSD post hoc test for analysis in time and 3-way ANOVA with LSD post hoc test for analysis of total values were performed. Number of animals in groups was: for 3 days, *n* = 19–26; for 5 days, *n* = 8–12; for 13 days, *n* = 9–16; for 27 days, *n* = 8–14.

Time	Group	Path	Locomotion	Resting	Rearing	Rearing	Rearing	Rearing
		Length	Time	Time	Time	Total	Supported	Free
		(cm)	(% of Time)	(s)	(s)	(Number)	(Number)	(Number)
3 DAYS	NS	222.1 ± 11.7	13.2 ± 0.8	9558 ± 833	91.1 ± 4.4	10.0 ± 0.8	4.9 ± 0.5	5.1 ± 0.4
	NL	111.6 * ± 15.8	4.9* ± 1.0	12,643 * ± 885	55.4 * ± 8.3	3.4 * ± 0.6	1.9 * ± 0.3	1.6 * ± 0.3
	KS	243.0 ^L^ ± 16.5	14.7 ^L^ ± 1.1	10,881 ± 731	61.9 * ± 3.9	8.5 ^L^ ± 0.9	4.8 ^L^ ± 0.5	3.6 *^,L^ ± 0.4
	KL	63.8 *^LK^ ± 13.2	2.6 *^,K^ ± 0.9	16,395 *^,L,K^ ± 1957	23.9 *^,L,K^ ± 6.0	0.8 *^,L,K^ ± 0.3	0.4 *^,L,K^ ± 0.1	0.3 *^,L,K^ ± 0.1
5 DAYS	NS	275.6 ± 17.6	16.2 ± 1.3	6334 ± 689	114.3 ± 8.9	12.5 ± 0.9	6.8 ± 0.7	5.6 ± 0.5
	NL	157.0 * ± 33.6	7.8 * ± 2.4	11,627 * ± 1009	110.7 ± 24.3	4.8 * ± 1.5	2.5 * ± 0.9	2.3 * ± 0.6
	KS	346.7 *^,L^ ± 35.1	20.8 *^,L^ ± 2.2	6459 ^L^ ± 667	79.8 ± 8.1	10.6 ^L^ ± 1.6	6.4 ^L^ ± 1.0	3.7 *^,L^ ± 0.4
	KL	116.5 *^,K^ ± 19.7	4.5 *^,K^ ± 1.0	13,814 *^,K^ ± 1781	56.7 *^,L,K^ ± 12.7	2.9 *^,K^ ± 0.9	1.4 *^,K^ ± 0.4	1.5 *^,K^ ± 0.6
13 DAYS	NS	246.7 ± 13.8	13.4 ± 1.0	6871 ± 526	125.4 ± 5.4	10.9 ± 0.9	5.9 ± 0.6	5.0 ± 0.3
	NL	213.4 ± 25.3	10.2 ± 1.8	6215 ± 692	168.1 * ± 17.1	10.7 ± 1.1	5.4 ± 0.8	4.3 ± 0.7
	KS	330.1 *^,K^ ± 22.5	20.3 *^,L^ ± 1.5	5285 ± 1044	101.7 ^L^ ± 9.0	10.7 ± 1.0	6.9 ± 0.8	3.6 *^,L^ ± 0.3
	KL	248.8 ^K^ ± 36.6	12.32 ^K^ ± 2.8	7575 ^K^ ± 1194	119.8 ^L^ ± 11.5	7.2 *^,L,K^ ± 1.2	4.4 ^K^ ± 0.7	2.3 *^,L^ ± 0.6
27 DAYS	NS	294.2 ± 19.2	17.2 ± 1.3	4868 ± 617	130.6 ± 8.0	14.3 ± 0.8	8.1 ± 0.6	6.2 ± 0.3
	NL	264.3 ± 31.7	13.7 ± 2.2	7705 * ± 1317	148.2 ± 11.4	12.2 ± 1.3	6.8 ± 0.8	5.4 ± 0.7
	KS	306.8 ± 23.6	18.4 ± 1.6	6616 ± 971	99.3 *^,L^ ± 6.6	9.6 * ± 0.9	5.6 * ± 0.7	4.0 * ± 0.3
	KL	346.3 ^L^ ± 45.1	18.9 ± 2.9	8360 * ± 1313	112.6 ^L^ ± 11.7	8.5 *^,L^ ± 1.7	4.9 * ± 0.9	3.6 *^,L^ ± 0.8

## Data Availability

The experimental data presented in this study are available on request from the corresponding author.

## Supplementary Materials

The following are available online at https://www.mdpi.com/article/10.3390/ijms22147556/s1.
