# Supplementary material for: Increased Beta-Hydroxybutyrate Level Is Not Sufficient for the Neuroprotective Effect of Long-Term Ketogenic Diet in an Animal Model of Early Parkinson’s Disease. Exploration of Brain and Liver Energy Metabolism Markers
Source: Int J Mol Sci. 2021 Jul 14;22(14):7556. doi: 10.3390/ijms22147556 (PMC8307513; doi:10.3390/ijms22147556)
Supplement: Supplementary file 1 [file ijms-22-07556-s001.zip › ijms-1230183-supplementary.pdf]

Supplementary Materials

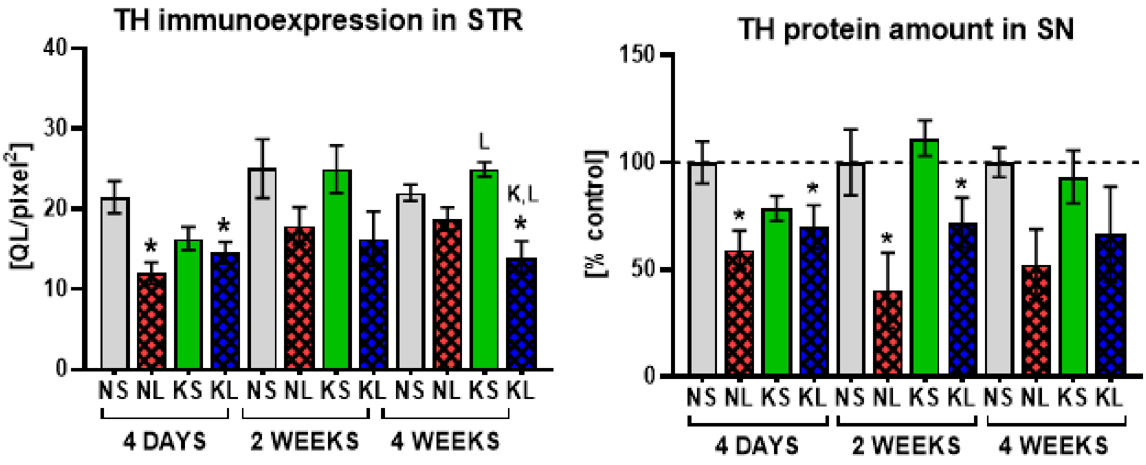

Figure S1. Tyrosine hydroxylase immunoexpression on tissue sections from striatum measured densitometrically and protein expression level measured in homogenates from the substantia nigra using Western blot. Data are presented as mean ± SEM. Analysis was performed at time-points 4 days, 2 weeks and 4 weeks after brain surgery. Animals were kept on ketogenic diet for 3 weeks in advance. *p* value ≤ 0.05 was set as significance threshold and marked as * vs normal diet + sham (NS group), L vs normal diet + 6-OHDA lesion (NL group), K vs ketogenic diet + sham (KS group), KL stands for ketogenic diet + 6-OHDA lesion group.

Table S1. Summary of published experimental data on ketogenic diet and ketone administration in animal models of PD.

PD Animal Model	Type of Diet/Substance	Glucose	Neuroprotection	Ref.
6-OHDA injected into MFB, 3µg, 8–16 weeks old Wistar rats	70% fat, 8% protein, 1% carbohydrates, 3 weeks prior lesion 4 weeks post lesion	no change	A tendency to increase locomotor activity, DA and its turnover normalization in STR, no neuroprotection	this research, Kuter et al. 2021
6-OHDA injected into striatum, 20µg, Wistar rats	90.7% fat, 9% protein, 0.3% carbohydrate, 2 weeks prior lesion 2 weeks post lesion	N/A	partially reversed decreases in DA neuron number, DA and its metabolites, GSH level	Cheng et al. 2009
6-OHDA injected into substantia nigra, 12µg 12–14 weeks old Wistar rats	50% fat, normal protein and carbohydrate, 11 days prior lesion 2 weeks post lesion	N/A	partially reversed decreases in cylinder and catalepsy tests	Shaafi et al. 2016
MPTP ip 4 times 20mg/kg, 8 weeks old C57BL/6J mice	70% fat, 20% protein, 0% carbohydrate, 1 week prior lesion 2 days/1 week post lesion	N/A	partially reversed decreases in DA neuron number, DA level, locomotion, microglia activation, pro-inflammatory cytokines	Yang and Cheng et al. 2010

MPTP ip 4 times 18mg/kg, 8–10 weeks old C57BL mice	normal diet B-HB infusion via osmotic pumps, 1 day before lesion for 7 days	N/A	partially reversed decreases in DA neuron number, DA and its metabolites level, locomotion	Tieu et al. 2003
MPTP ip 4 times 10mg/kg, 12-months old C57BL/10Tar mice	normal diet octanoic acid administration 1.5 hr before and for 2 consecutive days	N/A	Increased DA and metabolites in STR, increased mRNA of PGC-1α and PEPCK	Joniec-Maciejak et al. 2018
2'-methyl-MPTP 20mg/kg ip, 2 times, 10 week old male C57BL/6 mice	normal diet Pantethine 15 mg ip daily injection 5 days before, 5 days after lesion	N/A	partially prevented DA decrease, protected DA neuron and terminals loss and motility disorder, enhanced GSH production, restored CxI activity	Cornille et al. 2010 #

Cornille, E.; Abou-Hamdan, M.; Khrestchatisky, M.; Nieoullon, A.; De Reggi, M.; Gharib, B. Enhancement of L-3-hydroxybutyryl-CoA dehydrogenase activity and circulating ketone body levels by pantethine. Relevance to dopaminergic injury. *BMC Neurosci.* **2010**, *11*, 51, doi:10.1186/1471-2202-11-51.

Table S2. HPLC analysis of DA and 5-HT metabolites and turnover rates. Turnover rates were calculated as metabolite to neurotransmitter ratio. Data are presented as mean ± SEM. Analysis was performed at time-points 4 days, 2 weeks and 4 weeks after brain surgery. Animals were kept on ketogenic diet for 3 weeks in advance. *p* value ≤ 0.05 was set as significance threshold and marked as * vs normal diet + sham (NS group), L vs normal diet + 6-OHDA lesion (NL group), K vs ketogenic diet + sham (KS group), KL stands for ketogenic diet + 6-OHDA lesion group, 2W vs 2 week time-point, 4D vs 4 day time-point, nd – not detected. Statistics: 3 way ANOVA with LSD post hoc test. Number of animals was 5–7 per group.

SN		**DOPAC**		**HVA**		**3-MT**		**DOPAC/DA**		**HVA/DA**		**3-MT/DA**		**5HT**		**5HIAA**		**5HIAA/5HT**	
		Mean	± SEM	Mean	± SEM	Mean	± SEM	Mean	± SEM	Mean	± SEM	Mean	± SEM	Mean	± SEM	Mean	± SEM	Mean	± SEM
4 DAYS	**NS**	86.8	± 7.7	45.4	± 3.6	nd		14.6	± 0.4	7.8	± 0.5	nd		599.2	±46.1	230.0	±12.3	38.9	± 1.7
	**NL**	67.3	± 9.4	**29.9***	± 3.3	nd		**12.1***	± 0.7	**5.4***	± 0.2	nd		540.0	±18.9	233.5	±17.6	43.1	± 2.4
	**KS**	86.3	± 5.8	36.0	± 3.1	nd		**14.8**^{L}	± 0.9	**5.5***	± 0.7	nd		605.8	±27.6	251.8	±10.8	**45.2***	± 2.4
	**KL**	86.0	± 5.7	**31.9***	± 2.5	nd		13.7	± 0.3	**5.4***	± 0.4	nd		526.4	±33.0	241.4	±16.4	**46.0***	± 1.8
2 WEEKS	**NS**	70.0	± 8.3	**30.9** ^{4D}	± 3.9	nd		13.6	± 0.8	6.4	± 0.3	nd		543.5	±23.2	196.3	± 8.8	37.0	± 1.4
	**NL**	**21.7***^{4D}	± 4.6	**11.2***^{4D}	± 1.3	nd		**9.6***^{4D}	± 1.4	**5.0***	± 0.4	nd		533.0	±29.1	178.2	±12.3	**33.4**^{4D}	± 1.6
	**KS**	68.5	± 5.5	27.8	± 3.0	nd		**12.2**^{4D}	± 0.7	**4.4***	± 0.3	nd		594.2	±30.3	**222.2**^{L}	±10.4	**37.6**^{4D}	± 1.3
	**KL**	**7.9** *^{K4D}	± 2.3	**9.0***^{K4D}	± 0.5	nd		**6.4***^{LK4D}	± 1.1	**6.1**^{K}	± 0.7	nd		588.5	±28.5	**220.0**^{L}	± 6.4	**39.6**^{4D}	± 2.0

4 WEEKS	**NS**	**55.0**^{4D} ± 6.4	**25.7**^{4D} ± 1.6	nd	**11.5**^{4D} ± 0.9	**5.5**^{4D} ± 0.4	nd	560.1 ± 26.5	194.6 ± 7.5	35.1 ± 1.9
	**NL**	**24.5***^{4D} ± 6.5	**13.3***^{4D} ± 2.1	nd	**9.2**^{4D} ± 0.2	5.0 ± 0.7	nd	599.1 ± 15.9	**210.8**^{2W} ± 11.8	**35.2**^{4D} ± 1.7
	**KS**	65.0 ± 5.1	31.7 ± 3.8	nd	**13.7**^{L} ± 0.9	6.1 ± 0.7	nd	625.1 ± 36.8	223.3 ± 13.1	**33.0**^{4D2W} ± 2.1
	**KL**	**19.7***^{K4D} ± 5.0	**20.6**^{4D2W} ± 7.4	nd	**7.2***^{K4D} ± 0.7	6.1 ± 0.7	nd	601.9 ± 16.1	229.2 ± 17.9	**36.1**^{4D} ± 1.0

**STR**		**DOPAC**	**HVA**	**3-MT**	**DOPAC/DA**	**HVA/DA**	**3-MT/DA**	**5HT**	**5HIAA**	**5HIAA/5HT**
		Mean ± SEM	Mean ± SEM	Mean ± SEM	Mean ± SEM	Mean ± SEM	Mean ± SEM	Mean ± SEM	Mean ± SEM	Mean ± SEM
4 DAYS	**NS**	1056.0 ± 51.9	760.8 ± 45.7	324.4 ± 15.3	8.5 ± 0.2	6.1 ± 0.4	2.6 ± 0.1	361.1 ± 12.4	372.0 ± 17.1	103.2 ± 4.2
	**NL**	**556*** ± 132.4	**349.1*** ± 94.8	**231.7*** ± 51.5	10.3 ± 1.4	6.1 ± 0.7	**4.4*** ± 0.7	396.7 ± 16.7	352.0 ± 25.4	88.6 ± 4.5
	**KS**	**1079**^{L} ± 68.7	**654.7**^{L} ± 86.7	311.0 ± 14.6	8.2 ± 0.4	5.0 ± 0.6	**2.4**^{L} ± 0.1	350.2 ± 21.1	397.8 ± 27.2	**114.5**^{L} ± 7.9
	**KL**	**516.3***^{K} ± 62.5	306.5 ± 34.0	**232.9*** ± 15.2	9.9 ± 0.7	6.0 ± 0.5	**4.7***^{K} ± 0.5	369.0 ± 35.3	374.5 ± 18.7	**97.2**^{K} ± 5.4
2 WEEKS	**NS**	1006.0 ± 59.1	594.0 ± 48.1	332.7 ± 13.1	7.8 ± 0.4	4.6 ± 0.4	2.5 ± 0.2	387.7 ± 16.3	336.2 ± 17.7	**86.1**^{4D} ± 3.5
	**NL**	**213.2***^{4D} ± 31.2	**136.7***^{4D} ± 24.1	**106.4***^{4D} ± 23.7	9.7 ± 0.8	6.2 ± 0.9	**4.5*** ± 0.3	350.6 ± 26.2	**268.7***^{4D} ± 22.8	**76.7**^{4D} ± 3.0
	**KS**	**970.1**^{L} ± 45.4	**592.5**^{L} ± 58.8	**318.2**^{L} ± 14.9	7.2 ± 0.3	4.4 ± 0.5	**2.4**^{L} ± 0.1	363.1 ± 34.8	**323.6**^{4D} ± 16.7	**92.3**^{4DL} ± 6.6
	**KL**	**232.8***^{K4D} ± 86.1	**168.6***^{K} ± 48.7	**82.9***^{K4D} ± 30.6	**15.0***^{LK4D} ± 2.3	**10.4***^{LK4D} ± 2.3	**5.8***^{K} ± 1.0	317.3 ± 32.0	**303.7**^{4D} ± 24.7	**97.9**^{L} ± 5.5
4 WEEKS	**NS**	1048.0 ± 62.8	592.1 ± 46.8	298.4 ± 12.4	7.4 ± 0.5	4.2 ± 0.3	2.1 ± 0.1	369.3 ± 18.4	**296.9**^{4D} ± 9.8	**81.3**^{4D} ± 4.4
	**NL**	**273.3***^{4D} ± 84.6	**217.0*** ± 59.0	**162.7*** ± 50.7	7.7 ± 0.9	5.4 ± 0.4	**5.5*** ± 1.5	393.6 ± 21.4	322.5 ± 10.6	82.4 ± 2.5
	**KS**	**1037**^{L} ± 55.9	**622.4**^{L} ± 61.5	**301.5**^{L} ± 13.1	7.7 ± 0.4	4.6 ± 0.4	**2.2**^{L} ± 0.1	342.1 ± 10.8	**306.5**^{4D} ± 12.8	**90.0**^{4D} ± 4.6
	**KL**	**566***^{K2W} ± 171.2	**453.2**^{2W} ± 151.0	**196.8**^{2W} ± 50.6	**8.0**^{2W} ± 0.5	**6.3***^{K2W} ± 0.8	**3.0**^{L4D2W} ± 0.4	**316.8**^{L} ± 27.2	321.2 ± 30.0	92.6 ± 5.5
